# Improvement of autochthonous *Saccharomyces cerevisiae* by rapid laboratory evolution technique of genome shuffling

**DOI:** 10.1042/BSR20253121

**Published:** 2025-09-12

**Authors:** Ravichandra Hospet, Devarajan Thangadurai, Jeyabalan Sangeetha, Natália Cruz-Martins

**Affiliations:** 1 Department of Botany, Karnatak University, Dharwad, Karnataka, India; 2 Department of Environmental Science, Central University of Kerala, Kasaragod, Kerala, India; 3 Department of Medicine, Faculty of Medicine, University of Porto, Portugal; 4 Life and Health Sciences Research Institute (ICVS), School of Medicine, University of Minho, Braga, Portugal

**Keywords:** alcohol production, genome shuffling, *Saccharomyces cerevisiae*, strain amelioration, sugar tolerance

## Abstract

The process of selecting indigenous *Saccharomyces cerevisiae* strains as a starter culture for specific fermentation has several requisites, including the assessment of distinct parameters based on desirable and traditional enological criteria. In most wineries, commercial *S. cerevisiae* strains are used for wine fermentation. However, it is rare to find indigenous *S. cerevisiae* strains used in wine production, even though these isolates are better adapted to specific regions and are often preferred for producing local fruit wines. Here, the identification and characterization of indigenous *S. cerevisiae* were carried out by 28S rRNA sequencing, followed by Fourier transform infrared spectroscopy analysis for further confirmation. The strain improvement technique of genome shuffling was incorporated to ameliorate sugar tolerance and enhance alcohol production in the *S. cerevisiae* RHTD10 strain. As a result, it was observed that the improved strain from the third round of shuffling tolerated sugar stress of 30% and produced 10.14 ± 0.21% of alcohol, which is higher than the wild strain of 7.11 ± 0.22% alcohol.

## Introduction

Must (juice) transformation into fine wine is influenced by well-optimized fermentation processes, in which wine yeasts display a key responsibility. Until 1980, the importance and role of native yeasts in wine production were observed in a relatively simplistic way [1]. Later, the potential of native yeasts in wine fermentation was progressively considered because of their unique flavor profile and efficiency in reproducible regional wines. The prior-most important function of wine yeasts is to assure a quick and full utilization of sugars for the production of alcohol, carbon dioxide, and several secondary metabolites, ultimately avoiding the release of off flavors [2]. The alcohol percentage in produced wine varies with yeast strains used, and spontaneous fermentation has generally involved the activity of various yeasts dominated by *Saccharomyces cerevisiae* species [3]. However, the effects of spontaneous fermentation are frequently unpredictable, with some non-*Saccharomyces* species being able to produce unfavorable compounds. Generally, in the wine industry, there is a stablished proper control of the growth of undesirable yeast species, mostly through adding sulfur dioxide to the must and further inoculation of some selected *Saccharomyces* species, like *S. cerevisiae* [4,5].

*S. cerevisiae* strains involved in the fermentation process play a key role in wine’s features [6–8]. Most wine is produced using selected commercial *Saccharomyces* strains, despite several wine researchers and winemakers selecting autochthonous *S. cerevisiae* strains as starters. This selection of commercial dried yeasts, although common, significantly reduces the strains’ biodiversity undergoing natural fermentation [9]. On the other hand, given that autochthonous yeast isolates are not capable of competing with commercial wine strains, the incorporation of a strain improvement technique has shown to be of key importance. One of the main requirements in biotechnological exploration, namely with regard to the use of strain improvement programs, is to discover effective methods to improve microbial strains for the quality production of key metabolites, which will find several applications in the food industries [10]. Phenomenal developments in strain improvement technology lead to improving strains of industrially important microbes [11,12].

Generally, a conventional method is employed for yeast strain improvement, involving an asexual method that is affected by both sequential mutagenesis and screening procedures. As a consequence, a new microbial strain is mutagenized for developing a parental library of mutant populations. The screening of such microbes improves the selection of strains with a desirable phenotype. Nonetheless, this conventional procedure is found to be time-consuming [10,13,14]. At the same time, in the search for alternative efficient techniques, genome shuffling is conceived as a novel way of standardizing industrially important yeasts, especially important winery yeast strains. The most important feature of this technique is the ability to allow the shuffling of DNA by multi-parental crossing with whole genomes’ recombination, which is largely associated with protoplast fusion or other conventional breeding techniques [10,15]. There are a few key investigations aiming at improving wine yeasts using hybridization techniques studied by Coloretti et al. [16], Perez-Traves et al. [17], and Bellon et al. [18]. In comparison with hybridization breeding methods, genome shuffling is conceived as a more reliable and easy technique for ameliorating wine yeasts. In addition, this novel technique is cost-effective; the use of this microbial technique does not need any expensive facilities, is user-friendly, and can be applied in most laboratories [10]. We selected genome shuffling for this present study due to its proven ability to rapidly combine beneficial traits from diverse mutants and its non-GMO status, which is advantageous for applications in the food and beverage industry. Nevertheless, we acknowledge that genome shuffling is one of several effective strategies for yeast strain improvement. Alternative methods such as experimental evolution, adaptive laboratory evolution, CRISPR-based genome editing, and systems metabolic engineering have also been successfully used to enhance microbial traits. Compared to these approaches, genome shuffling offers a unique advantage by enabling multi-parental recombination across entire genomes, rapidly accumulating beneficial traits even without detailed genetic knowledge. While protoplast fusion can be technically demanding, this technique is cost-effective and particularly valuable when fast improvement is needed. Overall, genome shuffling remains a powerful and versatile tool for developing superior industrial yeast strains, and future integration with genomic analyses can further enhance its precision and impact.

The genome shuffling technique helps to ameliorate the complex phenotype of yeasts with industrial importance. Researchers described a novel, efficient method for genome shuffling to enhance ethanol production of *S. cerevisiae*. As yeast exhibits haplodiplobiontic life cycles, it favors sexual and asexual reproduction. The strains obtained after three cycles of shuffling not only enhanced resistance to ethanol-induced stress but also increased ethanol yield compared with the control [13]. In another study, efficient and rapid improvement of *S. cerevisiae* was reported. The investigation provides optimized conditions for the isolation of protoplast, cell inactivation, fusion, and regeneration [19]. These improved *S. cerevisiae* strains may not be obtainable through classical strain improvement methods and can further serve as a novel technique for the improvement of yeast isolates [20]. Protoplast fusion is one of the classic tools in strain improvement to obtain genetic recombinations and development of hybrid strains, thus being possible to create strains with desired features by using protoplast fusion throughout the genome shuffling process [10,21].

## Materials and methods

### Molecular characterization of *S. cerevisiae*

Autochthonous yeasts were isolated from the spontaneous fermentation of wild fruits. Yeast genomic DNA isolation and analysis were performed as per the standard protocol by Harju et al. [22] and Hanna and Xiao [23] with slight modifications. After two days of incubation at 30°C, yeast single colonies were picked from YPD (Yeast Extract Peptone Dextrose) plates and inoculated into 2 ml of rich YPD nutrient broth and incubated overnight at 30°C and subjected to DNA isolation. Further gel electrophoresis and NanoDrop analysis were followed for DNA quality for PCR amplification. PCR reaction buffer contained MgCl_2_ (15 mM) – 5 µl, dNTPs (5 mM) – 5 µl, forward primer (NL1) (5 pM) – 2.5 µl, reverse primer (NL4) (5 pM) – 2.5 µl, Taq DNA polymerase (1 U/µl) – 0.5 µl, DNA template – 1 µl, and PCR water – 33.5 µl. The 50-µl PCR reaction mixture was well mixed, and we kept the tubes in a thermocycler and performed the PCR reaction as per the standard cycling conditions as follows: step 1: 94℃ for 3 min (hot start), step 2: 94℃ for 30 s (denaturation), step 3: 55℃ for 30 s (annealing), step 4: 72℃ for 1 min 30 s (extension), step 5: 72℃ for 7 min (final extension), step 6: 12℃ for infinite (final hold), The amplification of the D1/D2 domain of LSU 28S rRNA gene sequencing was performed as per the Sanger sequencing protocol. The sequence obtained was then used for the identification of yeast using NCBI-BLAST analysis. The sequence was deposited in the NCBI database. Molecular Evolutionary Genetics Analysis (MEGA7) software was used for sequence alignment and inferring a phylogenetic tree.

### FT-IR analysis of autochthonous *S. cerevisiae*

Fourier transform infrared spectroscopy (FT-IR) works by passing a broad-spectrum infrared (IR) beam through a sample. Molecules absorb specific frequencies that are characteristic of their structure. The Fourier Transform converts the raw data (interferogram) into an interpretable IR spectrum. The culture incubated for 48 h at 30°C in YPD broth was centrifuged at 8000 rpm for 10 min. The pellet was thoroughly washed twice with 1X PBS solution. The pellet was conditioned for 3–4 h at −20°C and subjected to lyophilization. The lyophilized yeast was pelleted with potassium bromide, and informative windows in the spectrum were selected and combined to achieve optimal results. Furthermore, the transmission of the IR radiation across the yeast pellet was recorded in the range of 500–4000 cm^-1^ using a Bruker TENSOR II high throughput FT-IR instrument coupled with a pyroelectric deuterated lanthanum α alanine doped triglycine sulfate detector. The reference spectrum library was assembled fore further identification [24].

### Physiological and enological characterization

The autochthonous *S. cerevisiae* strains were subjected to screening for physiologically and enologically important parameters, such as temperature, ethanol, sugar, and sulfur dioxide tolerances. The YPD broth was used for the various tolerance studies [9]. The β-glucosidase activity was performed as per the standard protocol described by López et al. [25]. The basal media containing 1.7 g/l Yeast Nitrogen Base (Himedia), 5 g/l glucose, 5 g/l ammonium sulfate, and 20 g/l agar were prepared. After autoclaving, 2 ml of a sterile 1% (w/v) 4-methylumbelliferyl-β-D-glucopyranoside (Sigma-Aldrich) was added to 100 ml of medium and poured into sterile Petri plates. Yeast inoculum (24 h old) was point-inoculated onto the agar surface and incubated for four days at 28℃. The presence of enzymatic activity was assessed through the visualization of a fluorescent halo surrounding the yeast colony by exposing the plate to UV light. H_2_S production was evaluated by the method described by Comitini et al. [26]. H_2_S production was tested on Biggy agar (Himedia, India), which contains bismuth as an indicator. Following incubation during 4–6 days at 26℃, the colonies were evaluated as H_2_S negative (white colonies) or H_2_S positive (brown or dark brown).

### Genome shuffling of autochthonous *Saccharomyces cerevisiae*

The autochthonous *S. cerevisiae* strain was improved by the genome shuffling technique following the standard protocol by Cao et al. [27], Pinel et al. [20], Yin et al. [28] and Jingping et al. [19] with slight modifications. A loopful of culture was added to 10 ml of YPD broth and incubated overnight. Around 5 ml of culture was added to a fresh 15-ml medium (OD to 1). Cells were harvested by centrifuging at 4500 rpm for 15 min. The pellet was washed twice with Milli-Q water and 5 ml of hypertonic (HT) buffer. About 5 ml of buffer of yeast cells was irradiated with a TUV-15 W-254-nm lamp for 30 s at a distance of 20 cm, followed by 3% ethyl methanesulfonate (EMS) treatment for 30 min at 30℃, and the pellet was washed with HT buffer. Lysozyme (2%) was added for enzymatic digestion of the cell wall. The cell suspension was shaken at 100 rpm for 60 min at 28℃, and then protoplasts were washed twice with HT buffer. Inactivation of protoplasts was performed by subjecting them to a water bath (60℃) for 30 min and another part was irradiated by UV for 2 min under a 15-W UV lamp at a distance of 30 cm, and then centrifuged at 4000 rpm for 10 min. To the pellet, 5 ml of HT buffer containing 40% polyethylene glycol (PEG) was added and incubated for 60 min at 28℃ in a sample rotator and then centrifuged, and the pellet was washed with HT buffer. A phase contrast microscopy study was carried out to observe protoplast fusion. Cells were spread on regeneration media (RM), and cells that grew on RM will be considered as GS1 cells. Similarly, GS1 strains were fused with either of the parental strains randomly and regenerated to achieve maximum shuffling; the process was repeated for four cycles, and improved strains from each successive cycle were subjected to sugar tolerance and alcohol production.

### Batch fermentation process and synthetic grape must composition

Most of the wines are produced by either a batch or continuous fermentation process. The batch fermentation of wine is the process that starts with the addition of substrate and inoculum to the fermentor at time zero and ends with the final retrieval of fine wine [29]. The fermentation efficiency of autochthonous *S. cerevisiae* was analyzed in synthetic grape must by following the method described by Rossouw and Bauer [30] and Viana et al. [31]. The grape must is a complex matrix with remarkable impact throughout the whole process of wine fermentation, despite presenting a very variable composition; thus, for ensuring reproducibility, a synthetic grape must with a well-defined composition was selected. Briefly, glucose and fructose (115 g/l each) were used as carbon and energy sources. Citric (0.3 g/l), malic (4 g/l), tartaric (3 g/l), and gallic (2 g/l) acids, magnesium sulfate (200 mg/l), monopotassium phosphate (2 g/l), ammonium chloride (460 mg/l), potassium sulfate (500 mg/l), ascorbic acid (500 mg/l), inositol (100 mg/l), niacin (2 mg/l), arginine (700 mg/l), glutaric acid (500 mg/l), proline (500 mg/l), manganese (II) chloride tetrahydrate (200 µg/l), and zinc chloride (135 µg/l) were added as the essential grape must compositions. Ergosterol (15 mg/l) and sodium oleate (5 mg/l) were added as anaerobic growth factors, and the pH was adjusted to 3.5 using orthophosphoric acid. Potassium metabisulfite (100 ppm) was added, similar to standard enological treatments.

### Sugar tolerance and GC-FID analysis for estimation of alcohols

Sugar tolerance was evaluated by subjecting them to 30–40% of sugar stress [9]. Qualitative and quantitative estimation of alcohol was performed as per the method by Archana et al. [32]. Sample (2 ml) was taken in an Eppendorf tube and centrifuged at 8000 rpm for 10 min. The supernatant was collected in a fresh Eppendorf tube and again centrifuged at 8000 rpm for 10 min; the resulting supernatant was collected and filtered through a membrane filter of 0.45 µm PTFE filter paper. The filtrate was then used to perform gas chromatography (GC) analysis of alcohols. An alcohol standard was procured from Merck, India. Qualitative analysis of the alcohol was carried out using GC equipped with a flame ionization detector (FID) (Shimadzu, GC-2010). Zebron Wax plus capillary column containing PEG was used. The temperature program followed was 40℃ (1 min hold) to 70℃ at a rate of 5℃ min^-1^ and to 220℃ at a rate of 25℃ min^-1^ for 3 min. The carrier gas was nitrogen, and the flow rate was 1 ml/min; the injection volume was 0.5 µl.

## Results and discussion

### Molecular characterization of *S. cerevisiae*

**Figure 1:**
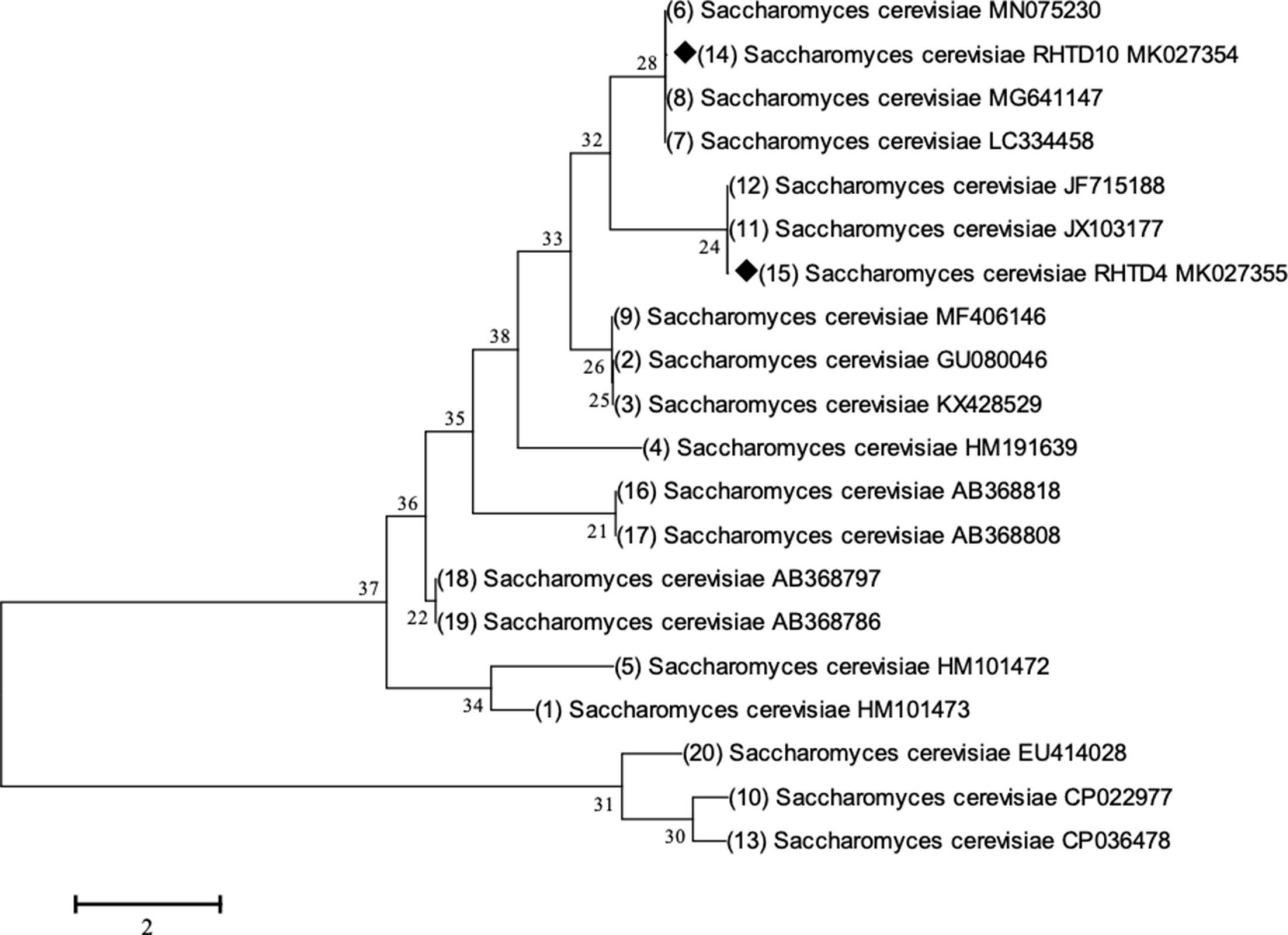
Phylogenetic relationship of autochthonous *S. cerevisiae* RHTD4 *and* RHTD10 with other *S. cerevisiae* species inferred using maximum likelihood method.

*S. cerevisiae* associated with fermented must was analyzed by molecular analysis through amplifying the D1/D2 domain of the LSU 28S rRNA gene sequence analysis [33,34]. The morphological and biochemical features were discussed in a previous study [35]. The standard yeast genomic DNA isolation protocol was followed, and the purified PCR product (100 ng) was used for sequencing. The sequence results were analyzed through the NCBI BLAST tool. The closest species co-ordinates with two test sequences were examined and identified as *S. cerevisiae* isolates, and the sequences were deposited to the NCBI database and got the accession number for *S. cerevisiae* RHTD4 – MK027355 and *S. cerevisiae* RHTD10 – MK027354, respectively. The evolutionary relationship was inferred using the maximum likelihood method. In the analysis, three major clusters and six sub-clusters depicted the evolutionary relationships among *S. cerevisiae* strains. The initial tree for heuristic search was retrieved by incorporating Neighbor-Join and Bio-NJ algorithms into the matrix of pairwise distances estimated by the maximum composite likelihood method and then selected the topology with superior log-likelihood value. The analysis involved 20 nucleotide sequences. All positions containing gaps and missing data were eliminated. There was a total of 577 positions in the final dataset. Evolutionary analyses were conducted in MEGA7 software [36,37](Figure 1).

### FT-IR analysis of *S. cerevisiae*

**Figure 2:**
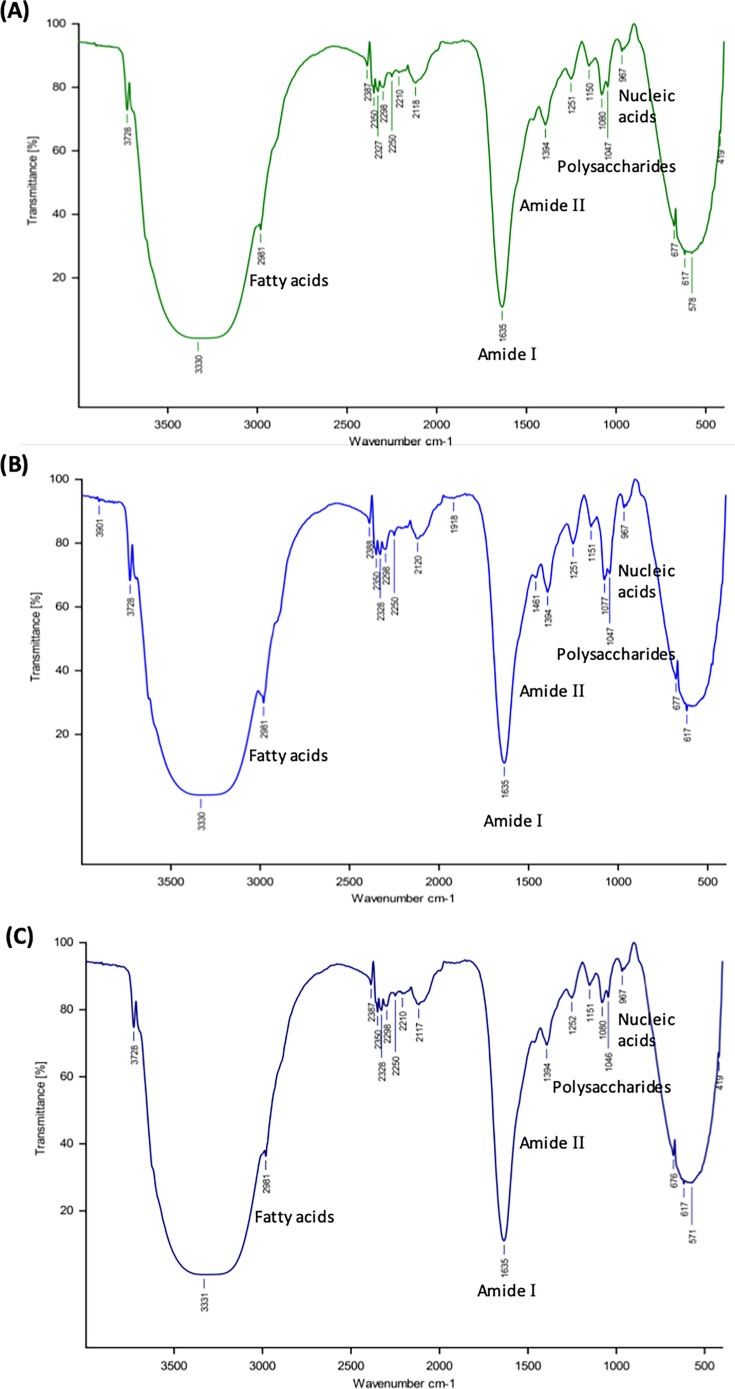
FT-IR spectrum of autochthonous *S. cerevisiae:* (**A**) RHTD4, (**B**) RHTD10, and reference strain NCIM 3215 (**C**) (French wine yeast).

The rapid and reliable method of FT-IR spectroscopy provides a high throughput technique for yeast identification [38]. The working principle of FT-IR corresponds to the fingerprinting technique; as each individual yeast exhibits distinct IR spectra, it was found to be a more reliable technique for yeast identification. The *S. cerevisiae* strains characterized by biochemical and molecular analysis were subjected to FT-IR-based analysis for further confirmation by comparing them with the known spectrum of reference strain NCIM 3215 – French wine yeast. The FT-IR spectra provided well-defined spectral regions corresponding to vibration of chemical entities and the yeast cell constituents. Absorption spectra with some characteristic spectral ranges were dominated by important chemical structures such as nucleic acid (700–900 cm^1^), polysaccharides (1200–900 cm^1^), amide II (1500–1600 cm^1^), amide I (1600–1700 cm^1^), fatty acids (3050–2800 cm^1^). Figure 2 shows the FT-IR spectrum of autochthonous *S. cerevisiae* RHTD4 and RHTD10 and reference strain NCIM 3215. Similar observations were reported by Kümmerle et al. [24] and Taha et al. [39] while standardizing rapid and reliable identification of indigenous yeasts by FT-IR spectroscopy.

### Assessment of physiological efficacy of *S. cerevisiae*

**Figure 3:**
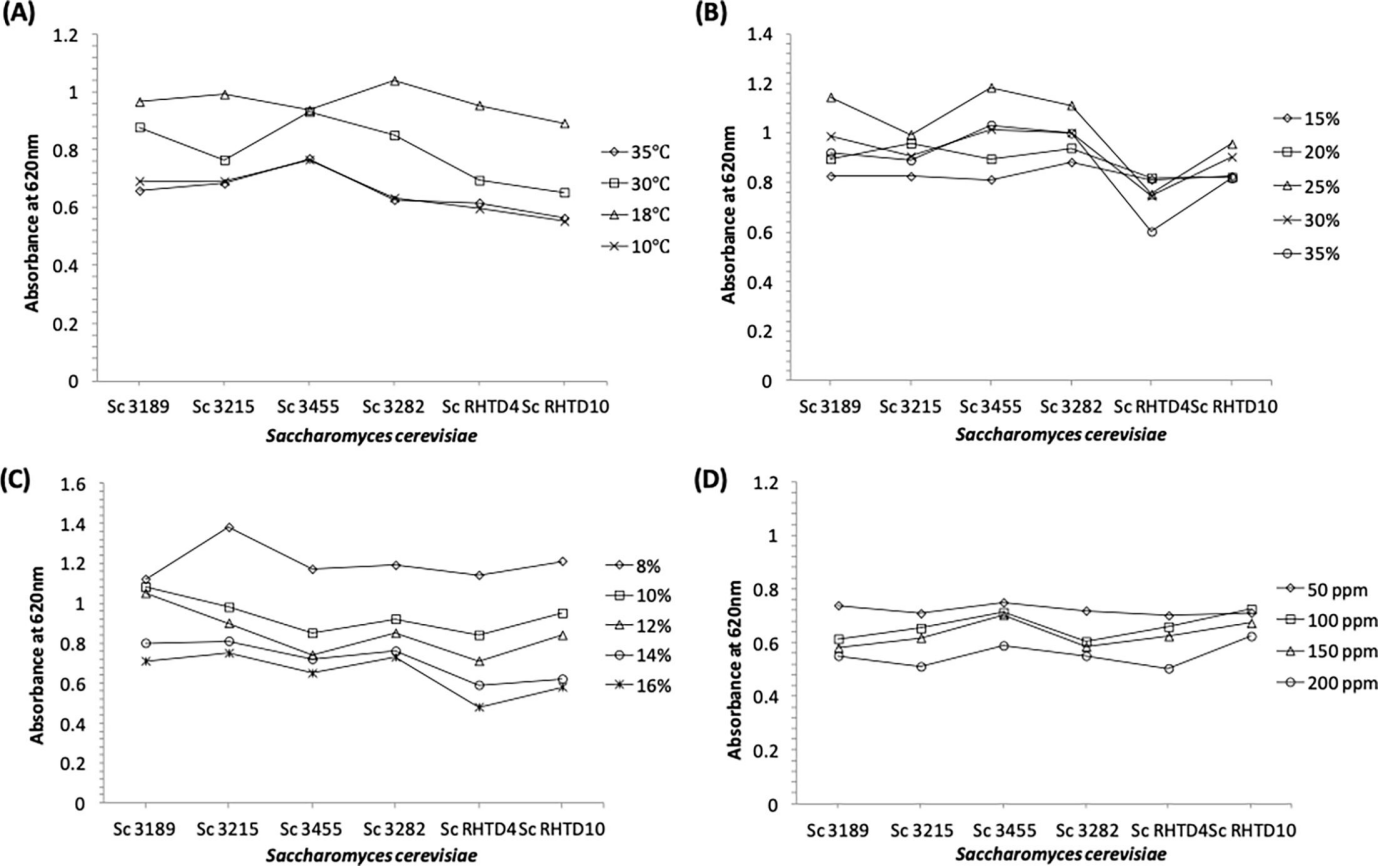
Physiological studies of autochthonous *S. cerevisiae:* (**A**) temperature tolerance, (**B**) sugar tolerance, (**C**) ethanol tolerance, and (**D**) sulfur tolerance.

*S. cerevisiae* RHTD4 and RHTD10 isolates were studied for their physiological ability along with four reference strains of *S. cerevisiae* (3189, 3215, 3455, and 3282) procured from the National Collection of Industrial Microorganisms (NCIM), National Chemical Laboratory, Pune. The strains were further subjected to various temperature tolerance (10–35℃), ethanol tolerance (8–16%), osmotolerance (15–35%), and sulfur dioxide tolerance (50–200 ppm K₂S₂O₅)(Figure 3). In temperature tolerance studies, *Sc* RHTD4 and *Sc* RHTD10 have shown good growth dynamics at 18℃, which is a very important observation made in the present study. This is an important feature for preserving fruitiness and aroma during fermentation. This particular temperature helps in the ideal wine fermentation process, mainly preserving fruitiness and volatile aroma compounds. Sulfur dioxide tolerance ability of *Sc* RHTD10 was observed to be the best among all the strains, a desirable trait for modern winemaking. In the sugar and ethanol stress test, *Sc* RHTD10 exhibits better tolerance than *Sc* RHTD4 but shows less tolerance when compared with reference strains. The β-glucosidase activity of autochthonous yeasts was carried out as per standard protocol. Moderate-to-low β-glucosidase activity was observed among the analyzed yeast strains. The yeast strains showed a medium-to-low H_2_S production on Biggy agar. Results are in good agreement with those reported by López et al. and Çelik et al. [40,41]. Similar observations were made while selecting autochthonous *S. cerevisiae* yeast for various types of wine production [42–45]. Since there is a great demand for autochthonous wine yeast starter cultures that are well adapted to the particular regions of the world with their respective fruit varietals, winemaking techniques, and types of wine, the present investigation provides an important step toward harnessing the enological potential of the untapped wealth of indigenous *S. cerevisiae* strains. It was observed that *Sc* RHTD10 was found to be better when compared with *Sc* RHTD4 strain during overall physiological and enological characterization. Further autochthonous *Sc* RHTD10 was deposited to the NCIM resource center, CSIR-NCL, Pune and received the accession number as 3680. Hence, *Sc* RHTD10 was selected for further strain improvement studies.

### Enhanced sugar tolerance and alcohol production

**Figure 4:**
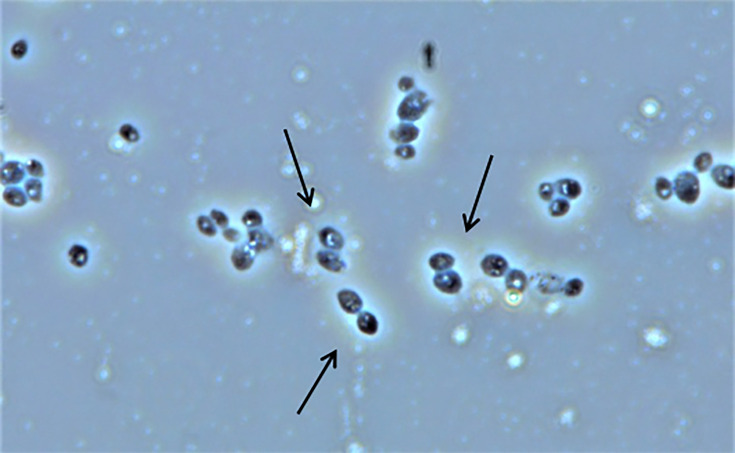
Phase-contrast microscopic image of protoplast fusion in *S. cerevisiae* strain RHTD10 GS3.

**Figure 5:**
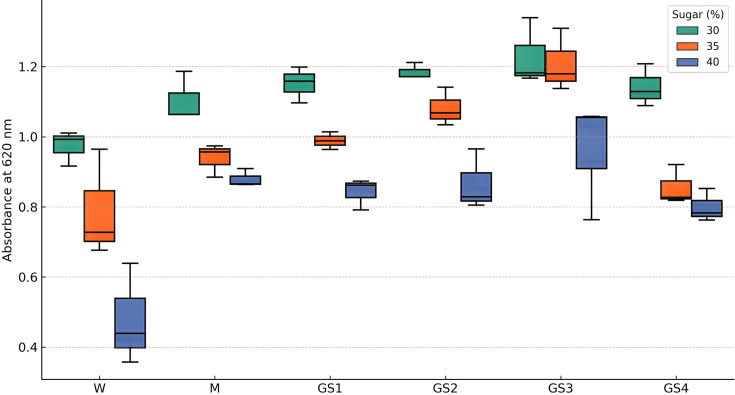
Evaluation of sugar tolerance efficiency of genome-shuffled *S. cerevisiae* strain RHTD10.

In the present study, a parental strain library was constructed using mutant autochthonous *Sc* RHTD10 strains by subjecting them to induce mutation. The classical approach toward the construction of the initial parental library is still in practice, such as direct selection and mutagenesis [10,46–48]. In the present study, EMS was used as a chemical mutagen and UV radiation as a physical mutagen. Yeast sexual and asexual life cycles favor shuffling of diploid mutant cells through rapid sporulation and extensive crossing among haploids. Despite various factors interfering in the recombination between yeast cells, it has been stated that protoplast fusion is a much more efficient method, resulting in high rates of gene transferability and proper recombination [10]. An elite protoplast fusion process, involving the recursive fusion of protoplasts within the multi-parents, provides high efficiency of fusion. The phenomena involved in recursive protoplast fusion are mixing, fusing, and regenerating protoplasts of the parental strains. Inactivated parental protoplast fusion is one such developed method that was used in genome shuffling. Generally, protoplasts were subjected to the irradiation process to achieve inactivation. If many parental protoplasts get inactivated by following the same process of inactivation, then it could be very difficult for the fused body to regenerate, thus being proposed the combination of inactivation methods, such as UV inactivation and heat shock treatment. In the present investigation, after cell lysis, the protoplasts isolated from *Sc* RHTD10 mutants were inactivated and fused in PEG medium and regenerated in specific regeneration media. Figure 4 depicts the phase-contrast microscopy of protoplast fusion. Strains obtained from rejuvenated protoplasts were accumulated and screened for sugar tolerance and alcohol production in synthetic grape media that resulted in a strain library for the next round of protoplast fusion. It was observed that there were good protoplast and regeneration frequencies in both regeneration and SDA (Sabouraud Dextrose Agar) media. Table 1 illustrates frequencies of protoplast formation and regeneration in *Sc* RHTD10. The subsequent strains from each round of shuffling were compared with wild and mutant strains (wild – W, mutant – M, first round shuffled strain – GS1, second round shuffled strain – GS2, third round shuffled strain – GS3, and fourth round shuffled strain – GS4) for sugar tolerance and alcohol production. The sugar tolerance ability of the strain was directly correlated with the increase in OD value at 620 nm (Table 2). It was observed that at the third round of shuffling, the growth of yeast was efficient and resulted in the highest tolerance of strain to sugar stress (30–40%). After the third round, the GS4 strain could not withstand the sugar stress but exhibited higher sugar stress than the wild strain (Figure 5).

**Table 1:** Frequencies of protoplast formation, regeneration in *S. cerevisiae* RHTD10

Genome shuffling rounds	cf.U/ml of initial cells (C)	cf.U/ml of cells on RM (A)^1^	cf.U/ml of cells on SDA (B)^2^	Protoplast formation frequency (A-B)/A	Regeneration frequency (A-B)/C
GS1	1.4 × 10^8^	2.5 × 10^7^	3.1 × 10^6^	8.7 × 10^-1^	1.5 × 10^-1^
GS2	1.2 × 10^9^	2.3 × 10^8^	3.3 × 10^7^	8.5 × 10^-1^	1.6 × 10^-1^
GS3	1.4 × 10^8^	2.4 × 10^7^	3.2 × 10^5^	8.6 × 10^-1^	1.4 × 10^-1^
GS4	1.6 × 10^8^	2.1 × 10^7^	3.1 × 10^6^	8.5 × 10^-1^	1.1 × 10^-1^

1 Colonies on RM plates from regenerated cells

2 Colonies on SDA plates after dilution of protoplasts with deionized water

**Table 2:** Sugar tolerance of *S. cerevisiae* strains (W, M, and GS1–GS4) at different sugar concentrations (OD at 620 nm)

Sugar (%)	W	M	GS1	GS2	GS3	GS4
30	0.933 ± 0.12ᵈ	1.08 ± 0.07ᶜ	1.12 ± 0.05ᵇ	1.19 ± 0.04ᵃᵇ	1.32 ± 0.08ᵃ	1.18 ± 0.09ᵃᵇ
35	0.818 ± 0.10ᵈ	0.97 ± 0.06ᶜ	1.01 ± 0.04ᵇ	1.10 ± 0.11ᵃᵇ	1.18 ± 0.07ᵃ	0.88 ± 0.05ᶜᵈ
40	0.612 ± 0.13ᵈ	0.85 ± 0.08ᶜ	0.88 ± 0.06ᵇᶜ	0.87 ± 0.09ᵇᶜ	1.01 ± 0.14ᵃ	0.81 ± 0.07ᶜ

Values are mean ± SD (n=3). Different superscript letters within a row indicate significant differences (*P*<0.05) based on Tukey’s test.

W, wild. M, mutant. GS1, first round shuffled strain. GS2, second round shuffled strain. GS3, third round shuffled strain. GS4, fourth round shuffled strain.

**Figure 6:**
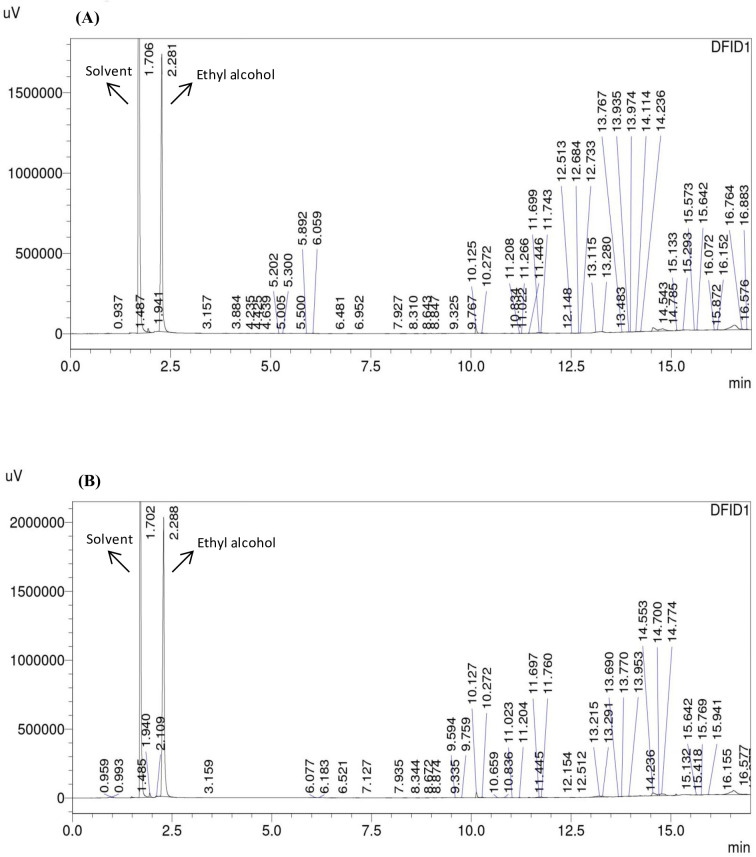
GC-FID analysis of alcohol contents in fermented synthetic media inoculated with *S. cerevisiae* strain RHTD10 wild (**A**) and *S. cerevisiae* strain RHTD10 mutant (**B**).

Similarly, alcohol production by all the strains was analyzed. Figures 6#x2013;8show the GC-FID chromatogram profile of alcohol (ethyl alcohol) content in fermented synthetic media inoculated with respective yeast strains, and it was observed that at the third round of shuffling, alcohol production was markedly enhanced in the GS3 strain, which achieved 10.14 ± 0.21% (v/v) alcohol, nearly threefold higher than the wild strain (7.11 ± 0.22%) and significantly higher than all earlier shuffling cycles (Figure 9 and Table 3). Similar potential abilities of yeasts were reported while improving various industrially important yeast strains. Ding et al. [49] resolved the problem of high concentrations of malic acids frequently observed in wines, which is detrimental to wine quality. The researchers have developed fusants of *Schizosaccharomyces pombe* through genome shuffling, which results in improved deacidification activity by minimizing the excessive malic acid content in *Vitis quinquangularis* Rehd wine. The application of genome shuffling in enological aspects was highly suggested by Giudici et al. [50]. The genome shuffling was used to improve the flavor profile of soy sauce by enhancing resistance to salt stress of *Zygosaccharomyces rouxii*. The researchers have enhanced the salt tolerance of *Z. rouxii* while simultaneously improving flavor formation in soy sauce. A mutant strain, S3‐2, with a high salt resistance was obtained after three cycles of shuffling [27]. While comparable to the other strain improvement techniques like classical methods and hybridization techniques, genome shuffling has more benefits of expressing complete genetic diversity. It is, thus, desirable to include combinatorial methods as an essential integral part of strain improvement technology. Compared with classical methods and hybridization, genome shuffling offers the advantage of rapidly recombining multiple beneficial traits and generating highly improved strains. Overall, genome shuffling proved to be a powerful, non-GMO, and cost-effective approach for improving RHTD10, resulting in a superior strain (GS3) with enhanced sugar tolerance and alcohol production. This present study highlights the potential of genome shuffling as an effective strategy to improve indigenous wine yeasts and supports its application for developing region-specific starter cultures with improved fermentation performance and quality attributes. The improvement of autochthonous *S. cerevisiae* of winery importance through genome shuffling technique was found to be unique, and probably the present investigation is one among the very few studies on wine yeast improvement through genome shuffling technique.

**Figure 7:**
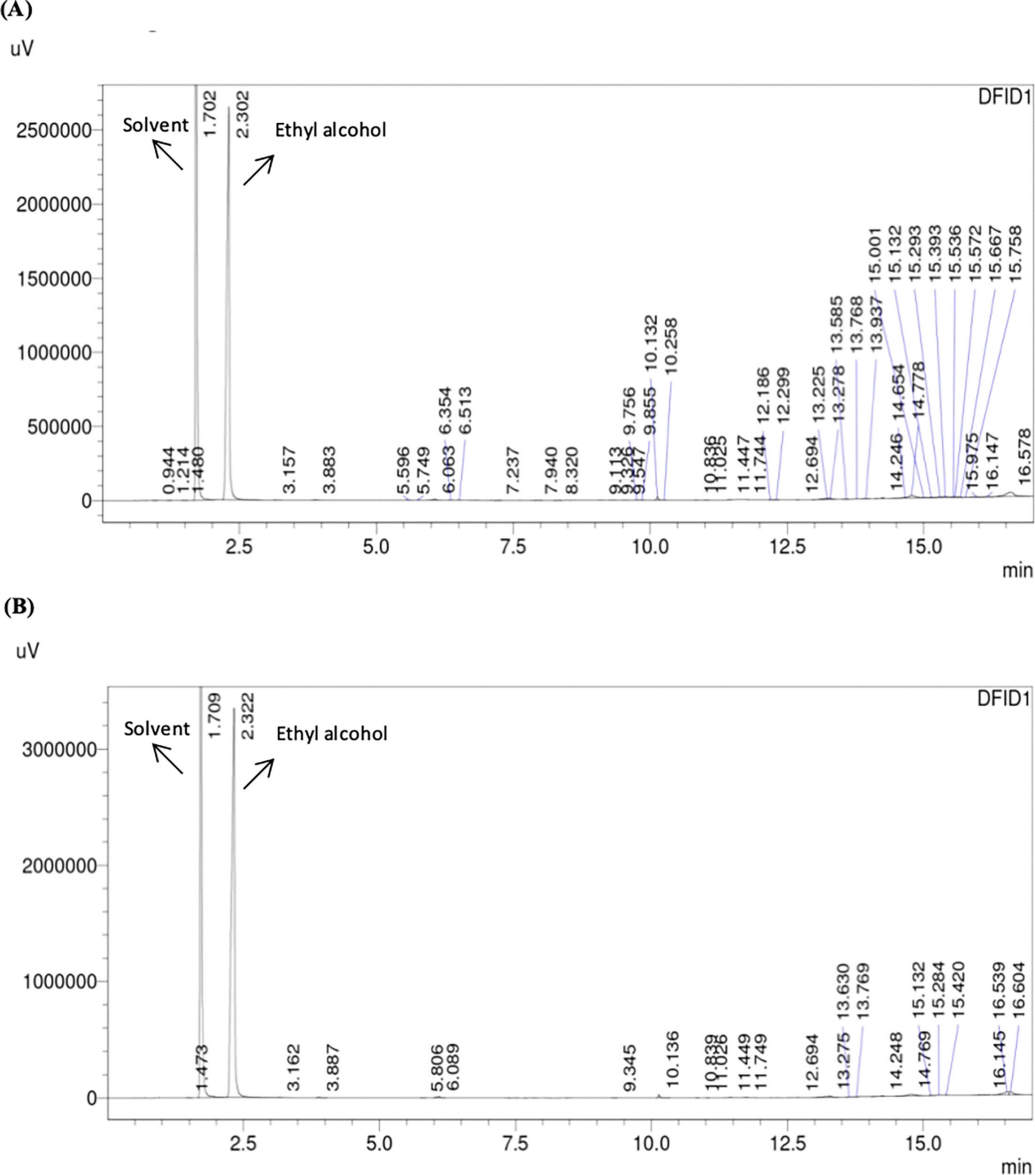
GC-FID analysis of alcohol contents in fermented synthetic media inoculated with *S. cerevisiae* strain RHTD10 GS1 (**A**) and *S. cerevisiae* strain RHTD10 GS2 (**B**).

**Figure 8:**
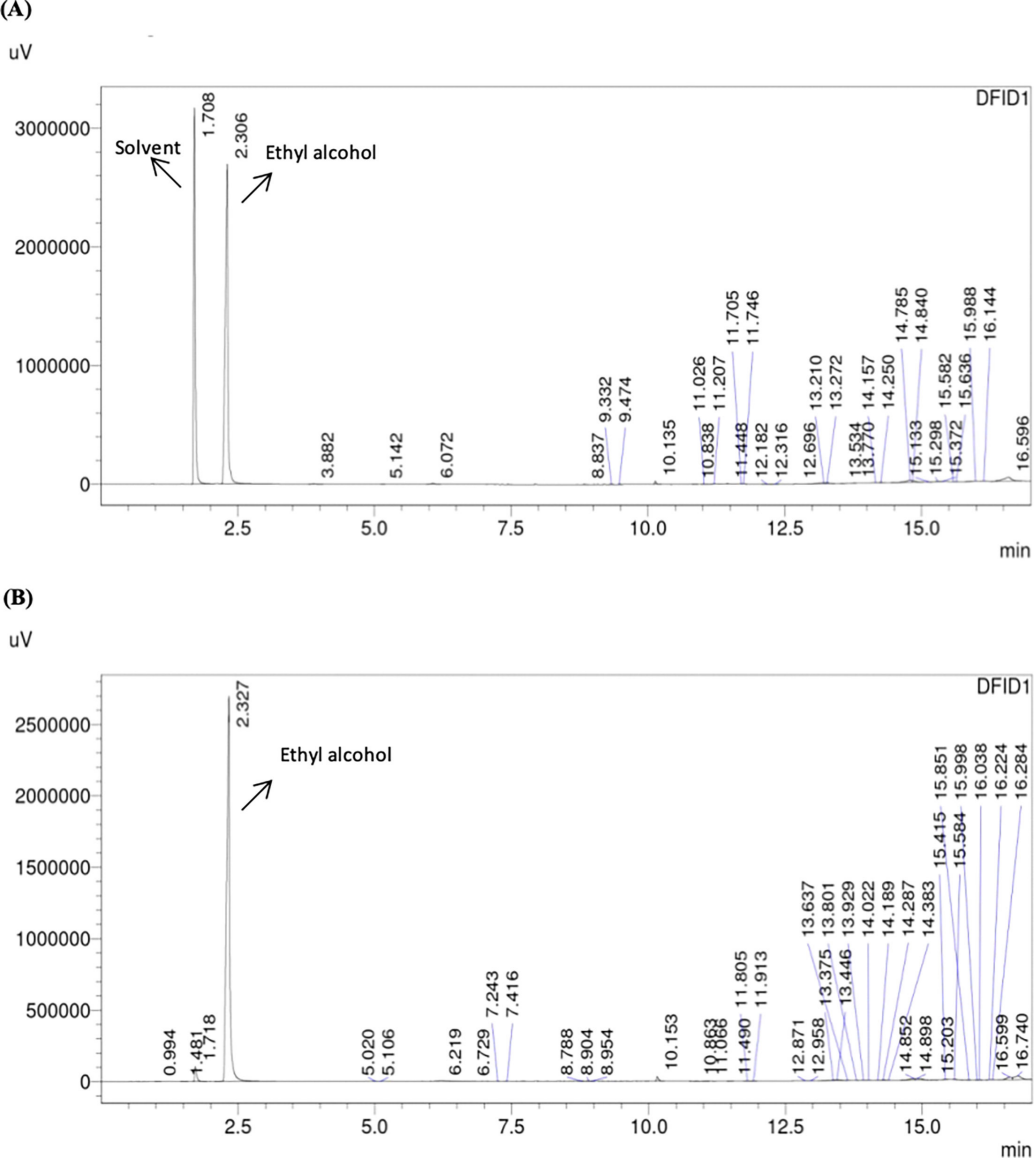
GC-FID analysis of alcohol contents in fermented synthetic media inoculated with *S. cerevisiae* strain RHTD10 GS3 (**A**) and *S. cerevisiae* strain RHTD10 GS4 (**B**).

**Figure 9:**
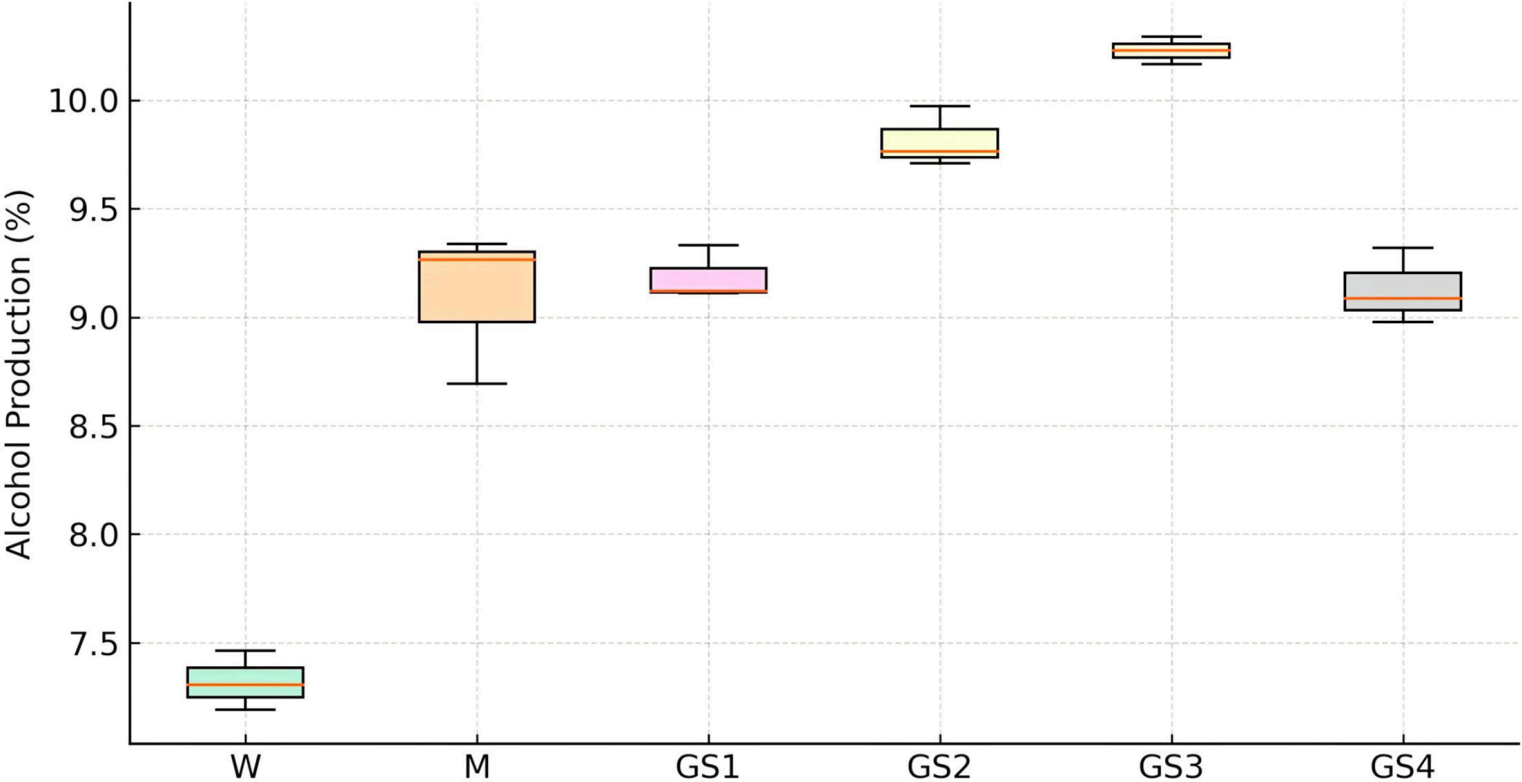
Alcohol production in fermented synthetic grape media inoculated with *S. cerevisiae* strain RHTD10 W, M, GS1, GS2, GS3, and GS4 strains. GS1, first round shuffled strain; GS2, second round shuffled strain; GS3, third round shuffled strain; GS4, fourth round shuffled strain; M, mutant; W, wild.

**Table 3:** Alcohol production by W, M, and GS1–GS4 *S. cerevisiae* strains.

Strain	Alcohol production (%) ± SD (Standard Deviation)
W	7.11 ± 0.22
M	8.89 ± 0.25
GS1	9.14 ± 0.18
GS2	9.68 ± 0.19
GS3	10.14 ± 0.21
GS4	9.02 ± 0.17

GS1, first round shuffled strain. GS2, second round shuffled strain. GS3, third round shuffled strain. GS4, fourth round shuffled strain. M, mutant. W, wild.

## Conclusion

The autochthonous *S. cerevisiae* strains were identified and characterized based on molecular, FT-IR, and enological features. Genome shuffling was used to ameliorate the sugar tolerance and alcohol production of *S. cerevisiae* RHTD10. The shuffled *S. cerevisiae* strain RHTD10 GS3 from the third cycle was observed to have great potential in sugar tolerance while producing the highest alcohol percentage among the tested strains in synthetic medium. The strain improvement through the genome shuffling technique highlighted new insights into the phenotypic improvement of autochthonous *S. cerevisiae* wine yeasts. This potential indigenous origin yeast strain can be further used for the production of various wines, and even those submitted to conventional methods can be subjected to genome shuffling for further quality improvement. Thus, genome shuffling can be viewed as a promising technique capable of improving multiple strains’ features.

## Data Availability

The datasets used and/or analyzed during the current study are available from the corresponding author upon reasonable request.

## Open Access

This article has been published open access under our Subscribe to Open programme, made possible through the support of our subscribing institutions, learn more here: https://portlandpress.com/pages/open_access_options_and_prices#conditional

## Abbreviations

EMS: ethyl methanesulfonate
FID: flame ionization detector
FT-IR: Fourier transform infrared spectroscopy
GC: gas chromatography
HT: hypertonic
IR: infrared
MEGA7: Molecular Evolutionary Genetics Analysis
NCIM: National Collection of Industrial Microorganisms
PEG: polyethylene glycol
